# Supplementary material for: Indirect SPECT Imaging Evaluation for Possible Nose-to-Brain Drug Delivery Using a Compound with Poor Blood–Brain Barrier Permeability in Mice
Source: Pharmaceutics. 2022 May 10;14(5):1026. doi: 10.3390/pharmaceutics14051026 (PMC9145277; doi:10.3390/pharmaceutics14051026)
Supplement: Supplementary file 1 [file pharmaceutics-14-01026-s001.zip › pharmaceutics-1681147-supplementary.pdf]

Supplemental materials

a)

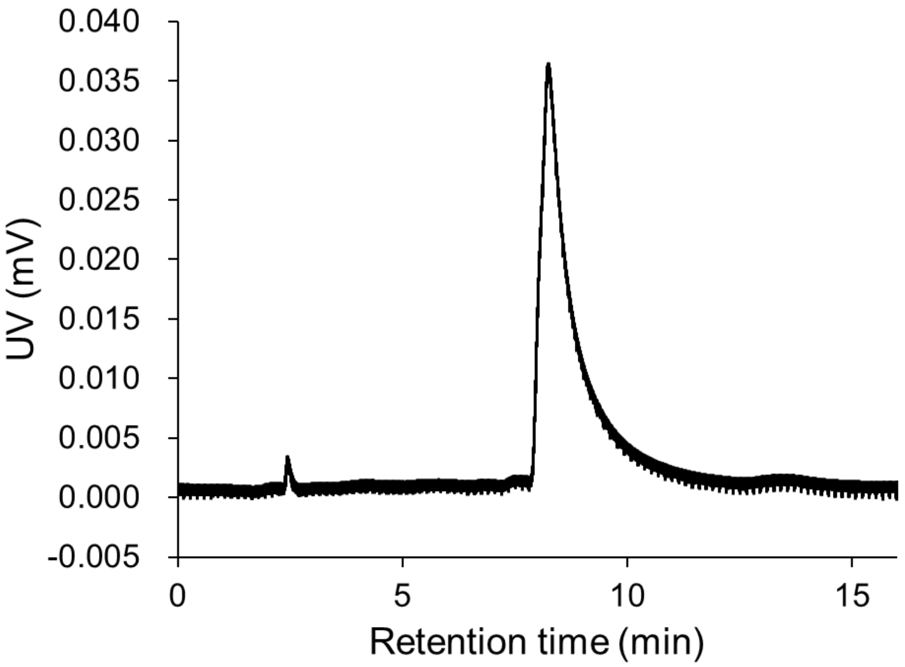

b)

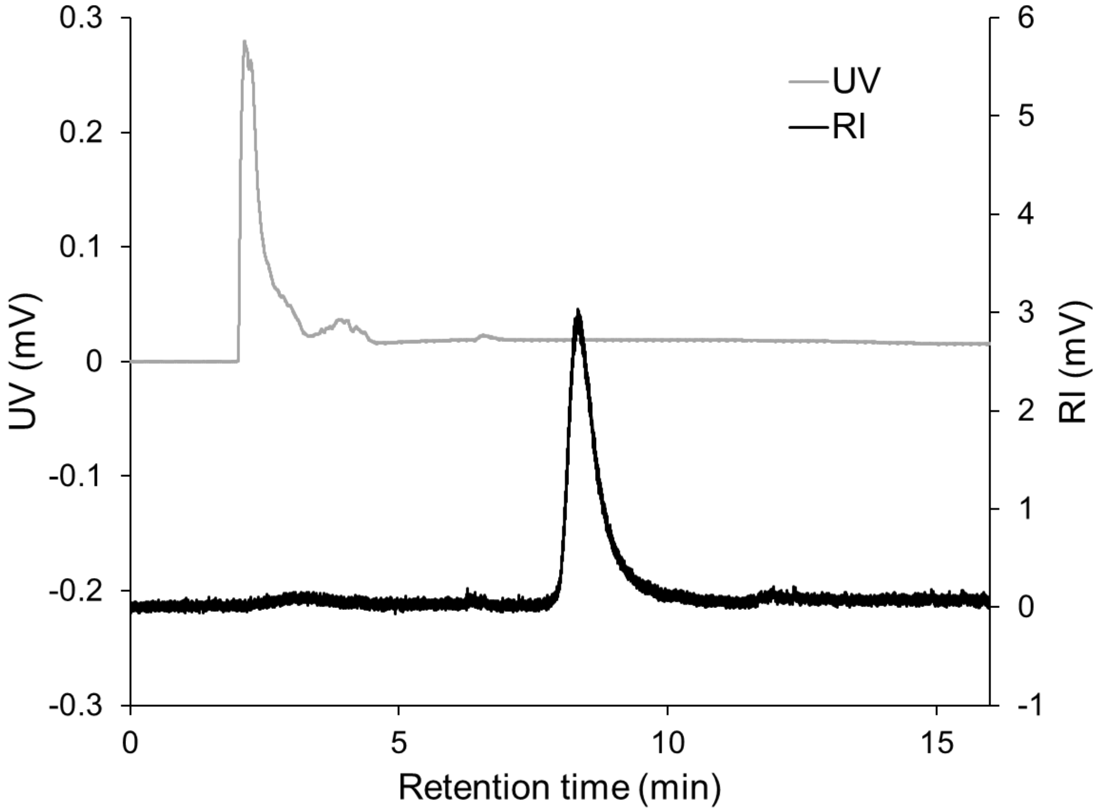

FIGURE LEGENDS

Supplemental materials

In the UV and radioisotope (RI) chromatograph, the peak waveform in UV of [^{127}I]IBZM (a) and RI of [^{125}I]IBZM (b) is confirmed at the same retention time of about 8.5 min.
